# Supplementary material for: Chloroplast Hibernation-Promoting Factor PSRP1 Prevents Ribosome Degradation Under Darkness Independently of 100S Dimer Formation
Source: Plants (Basel). 2025 Oct 13;14(20):3155. doi: 10.3390/plants14203155 (PMC12567329; doi:10.3390/plants14203155)
Supplement: Supplementary file 1 [file plants-14-03155-s001.zip › plants-3908188-supplementary.pdf]

Supplementary Materials

Article title: Chloroplast Hibernation-Promoting Factor PSRP1 Prevents Ribosome Degradation Under Darkness Independently of 100S Dimer Formation

Authors: Kenta Tanaka, Yusuke Yoshizawa, Takashi Oda and Yasuhiko Sekine

(A)

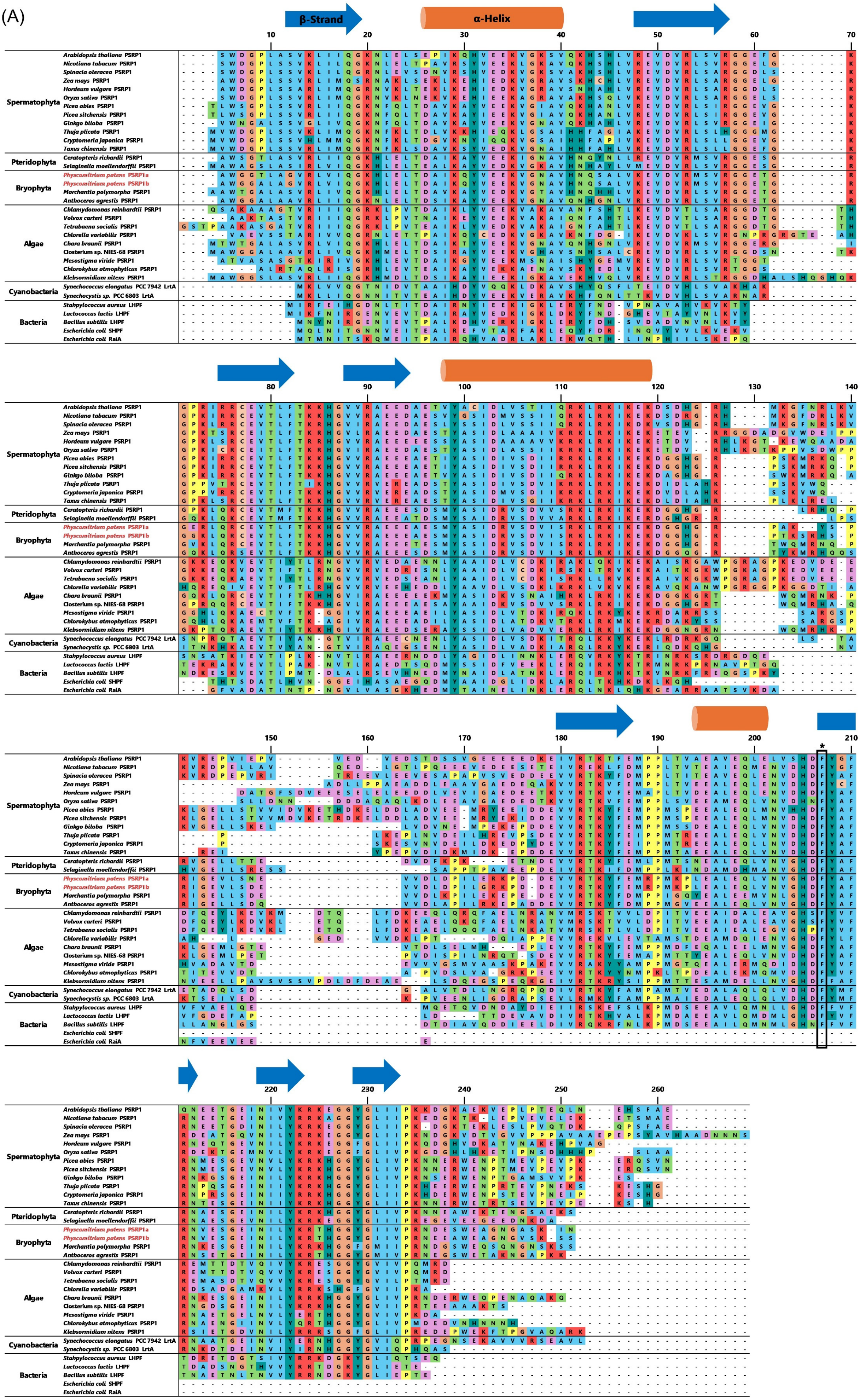
β-Strand
α-Helix
10
20
30
40
50
60
70
80
90
100
110
120
130
140
150
160
170
180
190
200
210
220
230
240
250
260
*
Spermatophyta
Pteridophyta
Bryophyta
Algae
Cyanobacteria
Bacteria
Arabidopsis thaliana PSRP1
Nicotiana tabacum PSRP1
Spinacia oleracea PSRP1
Zea mays PSRP1
Hordeum vulgare PSRP1
Oryza sativa PSRP1
Picea abies PSRP1
Picea sitchensis PSRP1
Ginkgo biloba PSRP1
Thuja plicata PSRP1
Cryptomeria japonica PSRP1
Taxus chinensis PSRP1
Ceratopteris richardii PSRP1
Selaginella moellendorffii PSRP1
Physcomitrium patens PSRP1a
Physcomitrium patens PSRP1b
Marchantia polymorpha PSRP1
Anthoceros agrestis PSRP1
Chlamydomonas reinhardtii PSRP1
Volvox carteri PSRP1
Tetrabaena socialis PSRP1
Chlorella variabilis PSRP1
Chara braunii PSRP1
Closterium sp. NIES-68 PSRP1
Mesostigma viride PSRP1
Chlorokybus atmophyticus PSRP1
Klebsormidium nitens PSRP1
Synechococcus elongatus PCC 7942 LrtA
Synechocystis sp. PCC 6803 LrtA
Stahpylococcus aureus LHPF
Lactococcus lactis LHPF
Bacillus subtilis LHPF
Escherichia coli SHPF
Escherichia coli RaiA

(B)

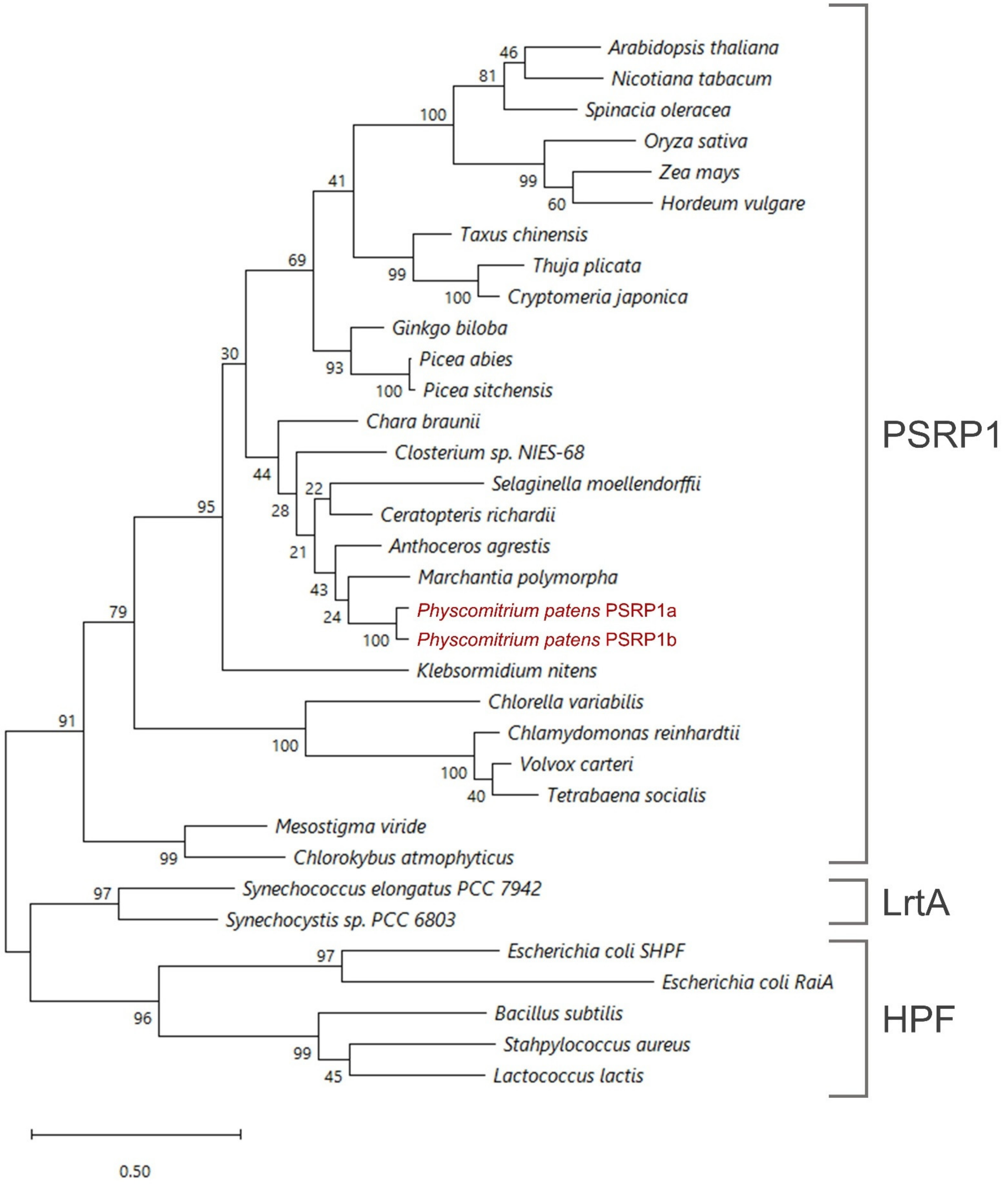
Arabidopsis thaliana
Nicotiana tabacum
Spinacia oleracea
Oryza sativa
Zea mays
Hordeum vulgare
Taxus chinensis
Thuja plicata
Cryptomeria japonica
Ginkgo biloba
Picea abies
Picea sitchensis
Chara braunii
Closterium sp. NIES-68
Selaginella moellendorffii
Ceratopteris richardii
Anthoceros agrestis
Marchantia polymorpha
Physcomitrium patens PSRP1a
Physcomitrium patens PSRP1b
Klebsormidium nitens
Chlorella variabilis
Chlamydomonas reinhardtii
Volvox carteri
Tetrabaena socialis
Mesostigma viride
Chlorokybus atmophyticus
Synechococcus elongatus PCC 7942
Synechocystis sp. PCC 6803
Escherichia coli SHPF
Escherichia coli RaiA
Bacillus subtilis
Stahpylococcus aureus
Lactococcus lactis
PSRP1
LrtA
HPF
0.50

(C)

```
P. patens PSRP1a   1 AWGGTLAGVRLVIQGKHLELTDAIKQYVEEKVGNAVHNQSALVKEVDVRM     50
                     ||||.|||||||||||||||||||||||||||||||||||||||||||||
P. patens PSRP1b   1 AWGGALAGVRLVIQGKHLELTDAIKQYVEEKVGNAVHNQSALVKEVDVRM     50

P. patens PSRP1a  51 SVRGGETGRGERLQRCEVTLFTKKHGVVRAEEEAESMYASIDRVSDVISR    100
                     ||||||||||.:||||||||||||||||||||||||||||||||||||||
P. patens PSRP1b  51 SVRGGETGRGGKLQRCEVTLFTKKHGVVRAEEEAESMYASIDRVSDVISR    100

P. patens PSRP1a 101 KLRKIKEKDGGHGRPAK--YSPRIGEVLSNEVVDLDPILERKPDDLPDEV    148
                     |||||||||||||||.|  :|||||||||:|||||||||.|||||||||:
P. patens PSRP1b 101 KLRKIKEKDGGHGRPTKSRHSPRIGEVLSDEVVDLDPILGRKPDDLPDEI    150

P. patens PSRP1a 149 VRTKYFEMRPMKPLEALEQLVNVGHDFYAFRNVESGEINILYKRTHGGYG    198
                     ||||||||||||||||||||||||||||||||||||||||||||||||||
P. patens PSRP1b 151 VRTKYFEMRPMKPLEALEQLVNVGHDFYAFRNVESGEINILYKRTHGGYG    200

P. patens PSRP1a 199 IIVPRNDESWEAGNGASKIN    218
                     ||||||:|:||||||.||.:
P. patens PSRP1b 201 IIVPRNEEAWEAGNGVSKSS    220    Percent Identity  94 %
```

Figure S1 Phylogenetic relationship among chloroplast PSRP1, cyanobacterial LrtA, and bacterial HPF proteins. (A) Amino acid sequences of chloroplast PSRP1, cyanobacterial LrtA, and bacterial HPF homologs were aligned using Clustal Omega multiple sequence alignment. The asterisk marks the position of a conserved phenylalanine residue (*Bacillus subtilis* numbering) that is essential for LHPF CTD dimerization. Chloroplast PSRP1 sequences from various species were used, including *Arabidopsis thaliana* (NP_568447.1), *Nicotiana tabacum* (XP_016459845.1), *Spinacia oleracea* (NP_001413380.1), *Zea mays* (NP_001151285.2), *Hordeum vulgare* (KAE8795426.1), *Oryza sativa* (XP_015630981.1), *Picea abies*, *Picea sitchensis* (ABR16805), *Ginkgo biloba*, *Thuja plicata*, *Cryptomeria japonica* (XP_057855523.1), *Taxus chinensis* (KAH9317356), *Ceratopteris richardii* (KAH7299333.1), *Selaginella moellendorffii* (XP_002975818.2), *Physcomitrium patens* (PSRP1a: XP_024359643.1, PSRP1b: XP_024402362.1), *Marchantia polymorpha* (OAE29788.1), *Anthoceros agrestis*, *Chlamydomonas reinhardtii* (XP_042924854.1), *Volvox carteri* (XP_002959239.1), *Tetrabaena socialis* (PNH05214.1), *Chlorella variabilis* (XP_005846155.1), *Chara braunii* (GBG87078.1), *Closterium* sp. NIES-68 (GJP29678.1), *Mesostigma viride*, *Chlorokybus atmophyticus*, and *Klebsormidium nitens* (GAQ78463.1). Cyanobacterial LrtA sequences from *Synechococcus elongatus* PCC 7942 (WP_011378421) and *Synechocystis* sp. PCC6803 (WP_010873244.1) were included. Bacterial HPF sequences from *Staphylococcus aureus* (WP_000617735), *Lactococcus lactis* (WP_011834629.1), *Bacillus subtilis* (WP_003228031.1), and *Escherichia coli* (SHPF: NP_417670, RaiA: NP_417088) were aligned as well. Chloroplast transit peptides at the N-terminus of PSRP1, predicted using TargetP 2.0 (https://services.healthtech.dtu.dk/services/TargetP-2.0/), were omitted to improve alignment accuracy. A schematic of the secondary structure based on predictions for *P. patens* PSRP1a and PSRP1b. PSRP1 sequences for *M. viride* (Gene ID: 6642) and *C. atmophyticus* (Gene ID: 593) were obtained from the Joint Genome Institute PhycoCosm, and for *G. biloba* (Gene ID: Gb_00693) from the Ginkgo database. *Picea abies* sequence (Gene ID: MA_165094g0010) was sourced from diurnal tools. For *T. plicata* and *A. agrestis*, sequences were retrieved via BLAST searches using *P. patens*

PSRP1a as the query from the Joint Genome Institute Phytozome and the Hornwort Genomes website, respectively. Other sequences were obtained from the NCBI and are followed by accession numbers after the species names.

(B) A maximum likelihood phylogenetic tree was constructed using Molecular Evolutionary Genetic Analysis (MEGA), with bootstrap values shown at the branches.

(C) Pairwise sequence alignment of PSRP1a and PSRP1b amino acid sequences, excluding the predicted N-terminal chloroplast transit peptides for improved accuracy, was performed using EMBOSS Needle.

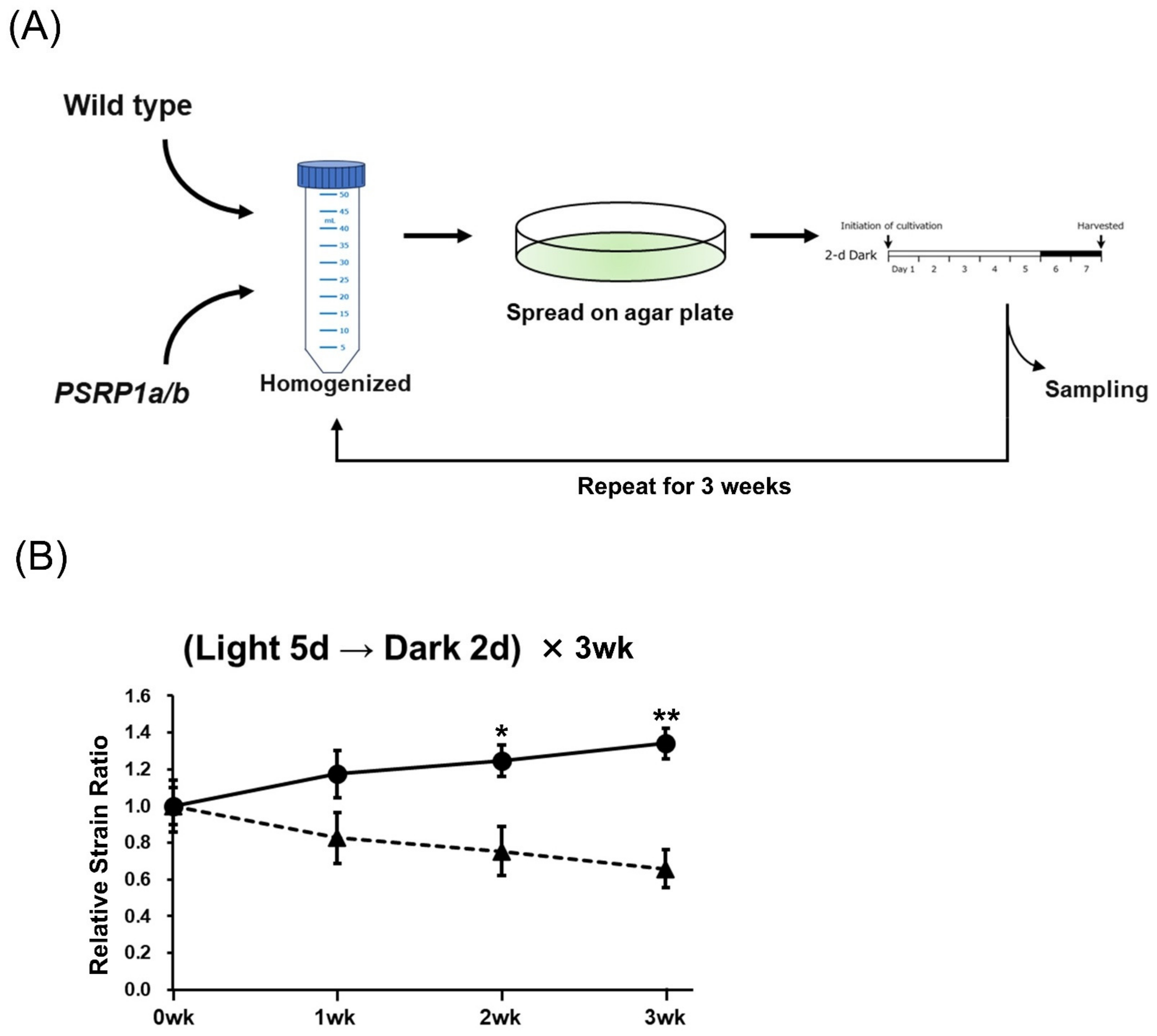

Figure S2 Growth comparison between wild-type and *PSRP1a/b* strains in competitive co-culture under prolonged dark conditions.

(A) Schematic overview of the assay. Wild-type and *PSRP1a/b* strains were mixed in water and spread on BCDAT agar plates, followed by co-cultivation under continuous light for 5 d and darkness for 2 d, for a total of 1 week. After 1 week, a portion of the cultures was sampled for analysis, and the remaining cultures were homogenized and grown again under the same conditions. This process was repeated for 3 weeks.

(B) The survival rates of wild-type and *PSRP1a/b* strain were calculated in the same manner as in Fig. 1C. The graph shows the average strain ratio ± standard error (n = 3). The solid line represents the wild-type strain, and the dotted line represents the *PSRP1a/b* strain. Significant differences after 3 weeks are indicated by asterisks (p < 0.05, Student's t-test).

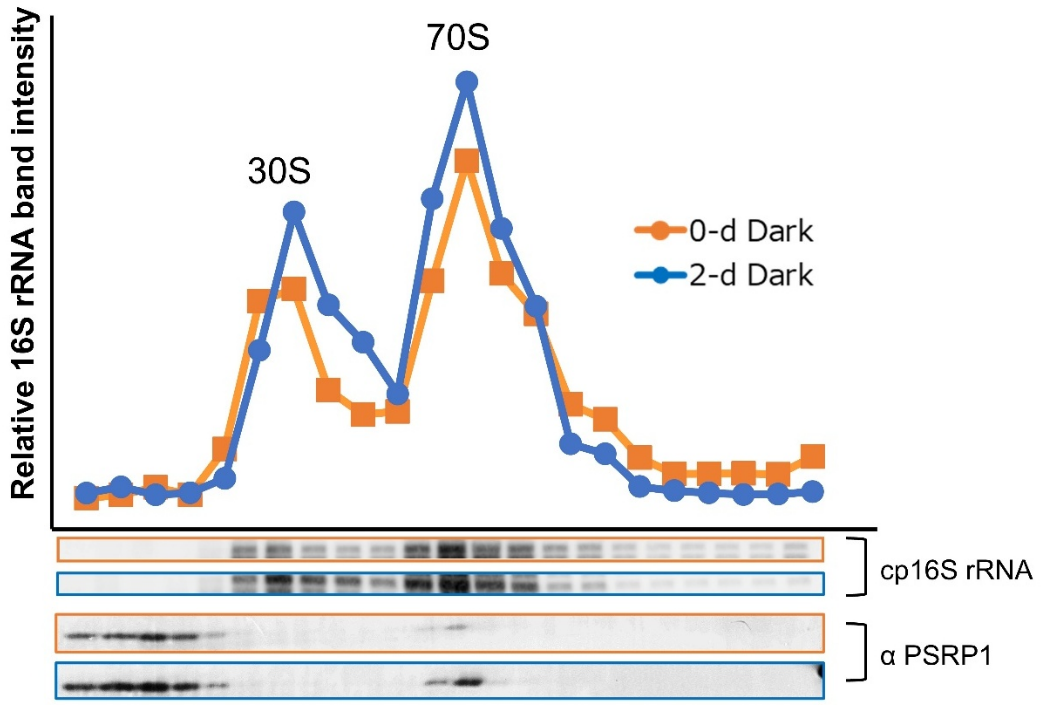

Figure S3 Polysome profiling in *P. patens* grown under 0-d or 2-d dark conditions.

Plant lysates were separated on 15–60% sucrose density gradients by ultracentrifugation. Total RNA was extracted from each fraction, and chloroplast 16S rRNA (cp16S rRNA) was detected by northern blotting using a specific probe against cp16S rRNA. The graph shows the quantification of cp16S rRNA based on the relative intensity of the band in each lane. Additionally, PSRP1 in each fraction was detected by western blotting using a specific antibody against PSRP1.

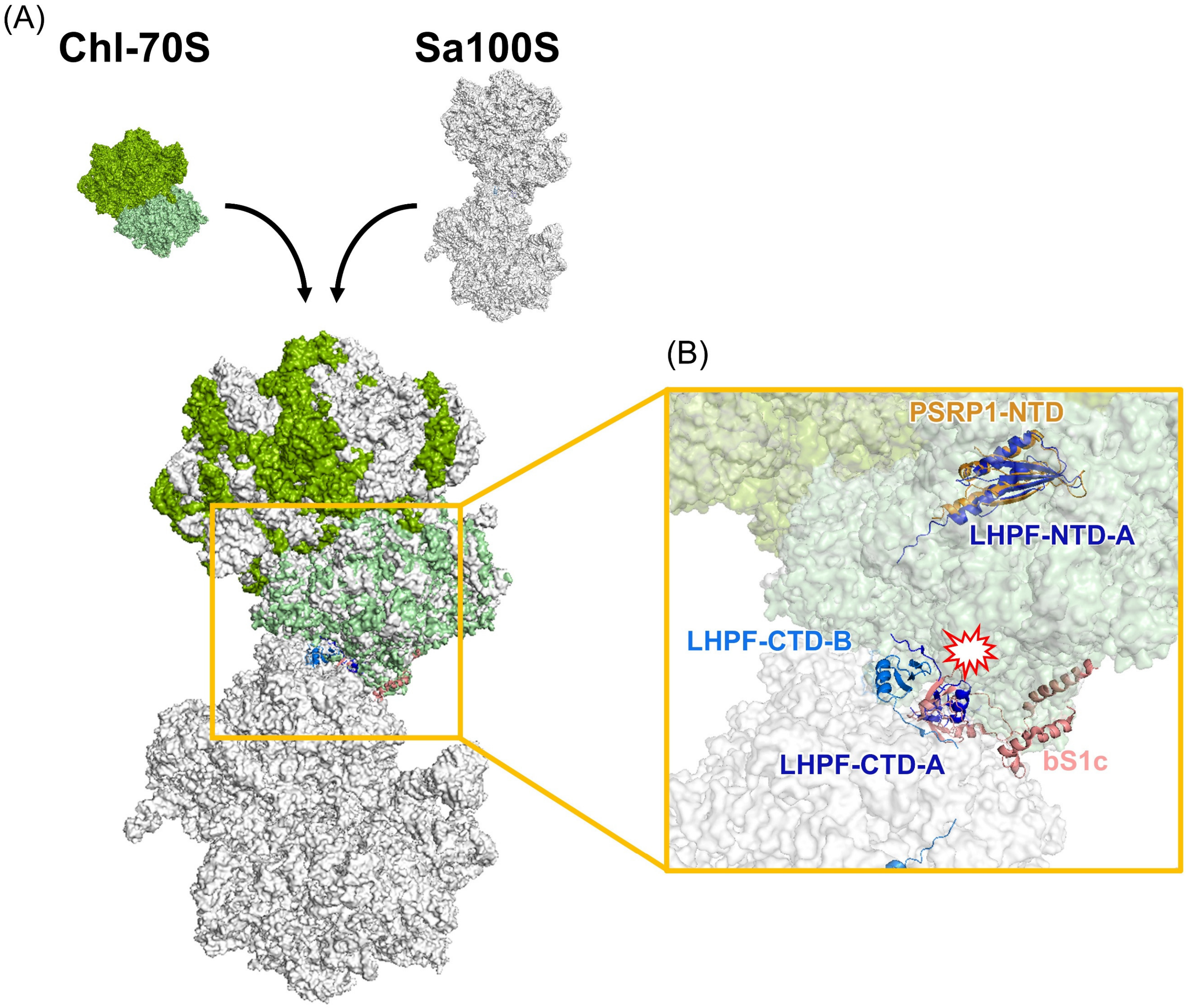

Figure S4 Structural comparison of the chloroplast ribosome and *S. aureus* 100S dimer.

(A) Superimposed structures of the spinach chloroplast 70S ribosome (PDB ID: 6ERI, green) and the *S. aureus* 100S ribosome dimer (PDB ID: 6FXC, grey).

(B) Close-up of the 30S-30S dimerization interface. In *S. aureus*, LHPF (blue) mediates dimerization via its C-terminal domain (CTD), while PSRP1 (brown) in the chloroplast ribosome shows a visible N-terminal domain (NTD), with the unresolved CTD likely due to its flexible nature. The chloroplast ribosome contains bS1c (light red) at the dimer interface, which may inhibit 100S dimer formation.

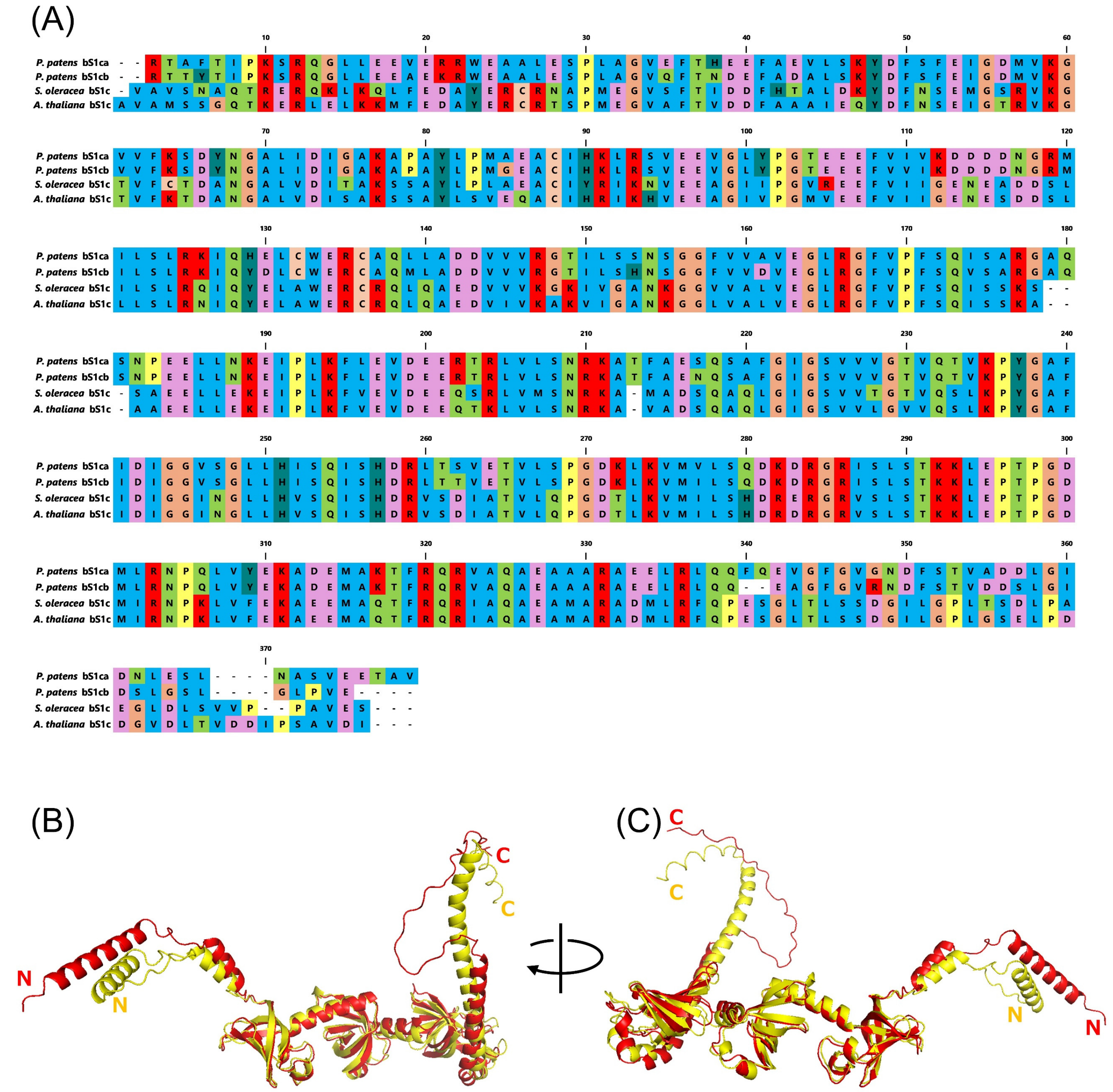

Figure S5 Sequence alignment and structural comparison of chloroplast bS1c proteins.

(A) Multiple sequence alignment of chloroplast bS1c proteins from *Physcomitrium patens* (XP_024356864.1, designated bS1ca; XP_024386702.1, designated bS1cb), *Spinacia oleracea* (NP_001413360.1), and *Arabidopsis thaliana* (NP_850903.1).

(B) and (C) Structural models of *P. patens* bS1ca (shown in yellow) and *S. oleracea* bS1c (shown in red) predicted by AlphaFold3 and superimposed. The two views represent the models from different orientations, with panel (C) showing a 180° rotation relative to panel (B).

Table S1 List of plasmids used in the study

Plasmid name	Description	Reference
pGFPmutNPTII	C-terminal sGFP fusion protein expression vector under native promoter in *P. patens*; Amp^R (*E. coli*), $G418^R$ (*P. patens*)	[54]
pGFPmutNPTII-PSRP1a	*PSRP1a* 5′/3′ HR (SalI/ClaI, XbaI/SacI) cloned into pGFPmutNPTII; Amp^R (*E. coli*), $G418^R$ (*P. patens*)	This study
pCTRN-NPTII 2	C-terminal citrine fusion protein expression vector under native promoter in *P. patens*; Amp^R (*E. coli*), $G418^R$ (*P. patens*)	NCBI Accession No. AB697058
pCTRN-aphIV	C-terminal Citrine fusion protein expression vector under native promoter in *P. patens*; Amp^R (*E. coli*), Hyg^R (*P. patens*). The nptII cassette in pCTRN-NPTII 2 was replaced with the *aphIV* gene from pTN186.	This study
pTaK16	C-terminal citrine knock-in vector for PSRP1b under native promoter in *P. patens*; Amp^R (*E. coli*), Hyg^R (*P. patens*). 5′/3′ HR from *P. patens* and fragments from pCTRN-aphIV were assembled with an NEBuilder® HiFi DNA Assembly Kit; Amp^R (*E. coli*), $G418^R$ (*P. patens*)	This study
pTN3	Knockout plasmid for replacing the target gene with a nptII cassette in *P. patens*; Amp^R (*E. coli*), $G418^R$ (*P. patens*)	[53]
pHAC7	Knockout plasmid for PSRP1a in *P. patens*, constructed by inserting the gene’s 5′/3′ flanking regions into pTN3 to replace the coding sequence with an nptII cassette; Amp^R (*E. coli*), $G418^R$ (*P. patens*)	This study
pTN186	Knockout plasmid for replacing the target gene with an aphIV cassette in *P. patens*; Amp^R (*E. coli*), Hyg^R (*P. patens*)	Addgene plasmid # 34890
pHAC8	Knockout plasmid for PSRP1b in *P. patens*, constructed by inserting the gene’s 5′/3′ flanking regions into pTN186 to replace the coding sequence with an aphIV cassette; Amp^R (*E. coli*), Hyg^R (*P. patens*)	This study
pET15b	*E. coli* vector for inducible expression of N-terminally 6xHis-tagged proteins; Amp^R (*E. coli*)	Addgene plasmid #69661-3
pTaK9	*PSRP1a* (*P. patens*) cDNA (NdeI/SalI) was inserted into pET15b (NdeI/XhoI); Amp^R (*E. coli*).	This study
pTaK10	*PSRP1b* (*P. patens*) cDNA (NdeI/BamHI) was inserted into pET15b (NdeI/BamHI); Amp^R (*E. coli*).	This study
pTaK34	*LlHPF* (*L. lactis*) DNA (NdeI/BamHI) was inserted into pET15b (NdeI/BamHI); Amp^R (*E. coli*).	This study

Table S2 List of oligonucleotides used in the study

Name	Sequence 5′-3′	Used in
PSRP1a RT-PCR Fwd	AGAGTGCTCTTGTAAAGGAAGTTG	RT-PCR
PSRP1a RT-PCR Rev	GTAACCTCACATCTTTGGAGTCTTT	
PSRP1b RT-PCR Fwd	CAGAGGTGTGAGGTCACATTATTC	RT-PCR
PSRP1b RT-PCR Rev	TGACATCGGAAACCCTGTCTAT	
PSRP1a-5′HR-SalI Fwd	cccc gtcgac TCCGCATTAGCTTTCGACTT	pGFPmutNPTII-PSRP1a
PSRP1a-5′HR-ClaI Rev	cccc atcgat GTTAATTTTTGATGCTCCATTG	
PSRP1a-3′HR-XbaI Fwd	tttt tctaga TTGTAAGCTTTTGTCCAGGGGG	
PSRP1a-3′HR-SacI Rev	gggg gagctc CTCACCCACAGGGTCAACAAAG	
aphIV Fwd	GCAAGACCCTTCCTCTATATAAG	pCTRN-aphIV
aphIV Rev	GATCTGGATTTTAGTACTGGATTTTGG	
pCTRN-NPTII 2 Fwd	CCAGTACTAAAATCCAGATC	
pCTRN-NPTII 2 Rev	ATATAGAGGAAGGGTCTTGCGAAGG	
PSRP1b-5’HR Fwd	TCCTCTTTTCAAGGTCAGGC	pTaK16
PSRP1b-5′HR Rev	GCTACTTTTCGATACTCCGT	
PSRP1b-3′HR Fwd	CTAGAGACATCTTTGTGGTC	
PSRP1b-3′HR Rev	GCCTTCATATATGCTAGCCA	
pCTRN-aphIV No.1 Fwd	acggagtatcgaaaagtagc GTCGACGGTATCGATAAGC	
pCTRN-aphIV No.1 Rev	gaccacaaagatgtctctag CGGCCGCTCTAGAACTAGTC	
pCTRN-aphIV No.2 Fwd	tggctagcatatatgaaggc CCACCGCGGTGGAGCTCCAG	
pCTRN-aphIV No.2 Rev	gcctgaccttgaaaagagga AGGGGGGGCCCGGTACCC	
PSRP1a-5′HR-KpnI Fwd	gag ggtacc GCAATGAGACATGGTATTGGGTAGTG	pHAC7
PSRP1a-5′HR-XhoI Rev	ggg ctcgag GTCCTACTCCTCTCCAACTCTGTCG	
PSRP1a-3′HR-SacII Fwd	ggg ccgcgg CGTCAGTGCCTAGAGTTCCACTGTA	
PSRP1a-3′HR-SacI Rev	ggg gagctc GGGTTGTGACAAACACAGTGAGCAT	
PSRP1b-5′HR-KpnI Fwd	gtg ggtacc GTTCCTCAGCTTTATCCTGATTGAAAGCC	pHAC8
PSRP1b-5′HR-XhoI Rev	ggg ctcgag ACGTCGTTACCCTCCCTTTCTGCAT	
PSRP1b-3′HR-SmaI Fwd	gag cccggg CCTTGAAAGTCTGGAGTTCCACTCTA	
PSRP1b-3′HR-SacI Rev	ggg gagctc GGCACTTTATAGCTTCGTACAACTACTAC	
PSRP1a-cDNA-NdeI Fwd	ggg catatg GCCTGGGGCGGTACTCT	pTaK9
PSRP1a-cDNA-SalI Rev	ggggg gtcgac CTAGTTAATTTTTGATGCTCCATTG	
PSRP1b-cDNA-NdeI Fwd	ggg catatg GCTTGGGGTGGCGCTCT	pTaK10
PSRP1b-cDNA-BamHI Rev	ggg ggatcc CTAGCTACTTTTCGATACTCCGTTACCAGC	
LlHPF-NdeI Fwd	ggg catatg ATGATCAAATTTAATATCCGTGGCG	pTaK34
LlHPF-BamHI Rev	ggg ggatcc TTATTCTGTTTCAATTAAGCCATAACGAC	
cp16S rRNA probe	GGTATTAGCAACCGTTTCCAGCTGTTGTCCCCCTCCCAA	Northern blotting
Cp23S rRNA probe	CCGGTGGGTTCCCTTAACCAGGCCACTACCTATAAGTCG	
PSRP1a genome qPCR Fwd	GCGGTGAAAGACTCCAAAGA	qPCR for PSRP1a
PSRP1a genome qPCR Rev	GCCAACCAGGACGAACTAAA	

NptII genome qPCR Fwd	CGTTGGCTACCCGTGATATT	qPCR for NPTII
NptII genome qPCR Rev	CTCGTCAAGAAGGCGATAGAAG	
P49 (Actin Fwd)	CAACCGTCTTCTGTGTCTAGGTC	qPCR for Actin [58]
P50 (Actin Rev)	GAAACCGGCCTGCATTACATG	
cp16S rRNA Fwd	CAGCTGGAAACGGTTGCTAATA	RT-qPCR for cp16S rRNA
cp16S rRNA Rev	TACTGATCGTCGCCTTGGTAA	
cp23S rRNA Fwd	GGAGAGAGACTCGGTGAAATAGA	RT-qPCR for cp23S rRNA
cp23S rRNA Rev	CCAATCCCAGGGAACAGTAAAG	
mt18S rRNA Fwd	TCAAACCAAGGATCACCCATTA	RT-qPCR for mt18S rRNA
mt18S rRNA Rev	CCACCTTGGTATCCCACATTAG	

Methods S1 Supplementary materials and methods

Phylogenetic analysis of plant PSRP1, cyanobacterial LrtA, and bacterial HPF

Protein sequences of plant PSRP1, cyanobacterial LrtA, and bacterial HPF were aligned using Clustal Omega [59] to generate multiple sequence alignments. The resulting alignment was analyzed using Molecular Evolutionary Genetic Analysis (MEGA) version 11 [60], with the maximum likelihood method to construct a phylogenetic tree.

Analysis of PSRP1 Binding to Chloroplast Ribosomes Using Sucrose Density Gradient Centrifugation

Sample preparation was performed as described in Section 4.7 of the main text. The supernatant was layered onto a 15–60% sucrose gradient prepared in gradient buffer (20 mM Tris-HCl, pH 7.5, 15 mM $Mg(OAc)_2$, 100 mM NH_4OAc, 6 mM β-ME) containing 0.2 mM PMSF and centrifuged at 21,000×g for 17.5 h at 4°C. The gradi

ent was fractionated using a Piston Gradient Fractionator™ (BioComp, Fredericton, Canada), and the absorbance at 254 nm was monitored using a Bio-mini UV Monitor (ATTO, Tokyo, Japan). Each fraction was used for western and northern blotting analyses.
